# Sustained attention operates via dissociable neural mechanisms across different eccentric locations

**DOI:** 10.21203/rs.3.rs-3562186/v1

**Published:** 2023-11-08

**Authors:** Tanagrit Phangwiwat, Phond Punchongham, Yodchanan Wongsawat, Itthi Chatnuntawech, Sisi Wang, Chaipat Chunharas, Thomas Sprague, Geoffrey F. Woodman, Sirawaj Itthipuripat

**Affiliations:** Department of Computer Engineering, King Mongkut’s University of Technology Thonburi; Department of Computer Engineering, King Mongkut’s University of Technology Thonburi; Department of Biomedical Engineering, Faculty of Engineering, Mahidol University; National Nanotechnology Center, National Science and Technology Development Agency; Department of Experimental and Applied Psychology, Vrije Universiteit Amsterdam; Chula Neuroscience Center, King Chulalongkorn Memorial Hospital, Thai Red Cross Society; Psychological and Brain Science, 251, University of California Santa Barbara; Department of Psychology, Vanderbilt University; Neuroscience Center for Research and Innovation (NX), Learning Institute, King Mongkut’s University of Technology Thonburi

**Keywords:** Attention, eccentricity, foveal vision, peripheral vision, EEG, SSVEP, visual cortex, frontoparietal cortex

## Abstract

In primates, foveal and peripheral vision have distinct neural architectures and functions. However, it has been debated if selective attention operates via the same or different neural mechanisms across eccentricities. We tested these alternative accounts by examining the effects of selective attention on the steady-state visually evoked potential (SSVEP) and the fronto-parietal signal measured via EEG from human subjects performing a sustained visuospatial attention task. With a negligible level of eye movements, both SSVEP and SND exhibited the heterogeneous patterns of attentional modulations across eccentricities. Specifically, the attentional modulations of these signals peaked at the parafoveal locations and such modulations wore off as visual stimuli appeared closer to the fovea or further away towards the periphery. However, with a relatively higher level of eye movements, the heterogeneous patterns of attentional modulations of these neural signals were less robust. These data demonstrate that the top-down influence of covert visuospatial attention on early sensory processing in human cortex depends on eccentricity and the level of saccadic responses. Taken together, the results suggest that sustained visuospatial attention operates differently across different eccentric locations, providing new understanding of how attention augments sensory representations regardless of where the attended stimulus appears.

## Introduction

The primate visual system comprises multiple cortical structures that are highly organized and interconnected (DeYoe and Van Essen, 1988; Felleman and Van Essen, 1991; Van Essen et al., 1992; Fox et al., 2005; Nassi and Callaway, 2009; Demirtaş et al., 2019). Neurons within these cortical structures form retinotopic maps, enabling simultaneous encoding and integration of visual inputs across the visual field (Engel et al., 1997; Serences and Yantis, 2006; Wandell et al., 2007; Sprague and Serences, 2013). To efficiently process fine-grained visual information, the visual system has developed a dual neural architecture, with higher temporal sensitivity in the periphery and higher spatial sensitivity in the fovea (Hartmann et al., 1979; McKee and Nakayama, 1984; De Valois and De Valois, 1988). This functional division allows humans to engage in tasks such as reading road signs while simultaneously monitoring the surrounding environment for vehicles and pedestrians.

To navigate through the environment, the visual system also requires selective attention to prioritize sensory inputs that are most relevant to behavioral goals. Although the differences in neuroanatomy between central and peripheral vision have been well-documented, it is still debated whether attention operates through the same or different neural mechanisms across eccentricities. A study of attention in nonhuman primates proposed that attention operated through different neural mechanisms across the foveal and peripheral locations (Roberts et al., 2007). Specifically, they found that attention increased the size of receptive fields (RFs) and the attentional gain of neurons in the primary visual cortex, whose RFs overlapped with attention and visual stimuli located at peripheral locations (Roberts et al., 2007). In contrast, reduced RF size and negligible attentional gain were observed near the fovea (Roberts et al., 2007). These findings suggest that the effects of attention on visuocortical processing are not uniform across eccentricities, and that foveal and peripheral attention may involve distinct cortical mechanisms, necessitating varying levels of perceptual demands and spatial integration of sensory information.

However, a later human EEG study found contradictory results, where a significant gain amplification of the early sensory response measured at the parafoveal locations was observed (Frey et al., 2010). They used this evidence to argue that foveal and peripheral attention operated via similar neural gain amplification mechanisms (Frey et al., 2010). However, this EEG study did not systematically examine the degree of attentional modulations as a function of eccentricity, calling into question whether the early sensory evoked responses undergo the same or different levels of attentional modulations across visual space.

Further complicating matters, fMRI studies have reported inconsistent results (e.g., Bressler et al., 2013; Sprague and Serences, 2013). In one study, the degree of attentional modulations of hemodynamic responses measured in early visual areas was much greater at the fovea compared to the more peripheral locations (Bressler et al., 2013). In contrast, in another fMRI study, opposing results were observed (Sprague and Serences, 2013). Specifically, in this study, the degree of attentional modulations on the amplitude of spatially selective sensory representations, based on the pattern of fMRI activity measured in early visual areas, did not change as a function of eccentricity (Sprague and Serences, 2013). These discrepant fMRI results could be due to eye movements since they were not monitored in these studies. This is highly likely because past studies have shown that eye movement control is supported by the prefrontal network and can cause changes in neural activity in up-stream visual areas (Chen et al., 2018; Noudoost et al., 2014; Schafer and Moore, 2011; Noudoost and Moore, 2011; Armstrong et al., 2006; Armstrong and Moore, 2007).

It is important to note that attentional modulations in fMRI signals in the early visual cortex reflect top-down synaptic inputs from the upstream attentional control regions to downstream visual areas and do not track the interaction between attention and sensory evoked responses or spiking outputs from the early visual areas per se (cf. Logothetis, 2002, 2008; Logothetis and Wandell, 2004; Viswanathan and Freeman, 2007; Itthipuripat et al., 2019b). Consistent with this idea, previous fMRI studies have shown that attention enhances fMRI activity in early visual areas even without the presence of a visual stimulus, and the degree of attentional modulations of fMRI activity is independent of stimulus intensity (Kastner et al., 1999; Buracas and Boynton, 2007; Murray, 2008; Pestilli et al., 2011; Hara and Gardner, 2014; Sprague et al., 2018; Itthipuripat et al., 2019). Thus, in addition to the contradictory findings in the fMRI literature, it remains unanswered whether the same or different neural mechanisms underlie the effects of selective attention on early sensory processes that occur at different eccentric locations.

Here, we aimed to distinguish between the competing accounts of the operation of attention mechanisms across different eccentric locations in the visual field. To do so, we monitored attention-induced changes in two EEG markers commonly used to index visual information processing, while human subjects attended to visual stimuli presented at different eccentric locations (i.e., attend-stimulus) or attended at the central fixation (i.e., attend-fixation). These EEG markers were the steady-state visually evoked potential (SSVEP) and the sustained negative deflection (SND) in the event-related potential (ERP), thought to track early sensory and top-down attentional control processes, respectively. The attentional modulations of the SSVEP signals are thought to index the attentional gain enhancement of the synchronous stimulus-evoked responses generated from the early visual areas (Regan, 1989; Norcia et al., 2015). On the other hand, the SND, which is the slow negative-going ERP wave, has been proposed to measure the top-down control signals from the fronto-parietal cortex onto the upstream visual areas (Grent-’t-Jong and Woldorff, 2007; Kelly et al., 2009; Itthipuripat et al., 2018, 2019; Hakim et al., 2019). To control for task difficulty, we equated accuracy levels across different attention tasks (i.e., attend-stimulus vs. attend-fixation) and different eccentricity levels. We also monitored eye movements via electrooculography (EOG) and sorted trails based on the levels of eye movements in order to examine the effects of eye movement on the patterns of attentional modulations of the SSVEP and SND data.

With a minimal level of eye movements, we found that the degree of attentional modulations of the SSVEP and SND responses were heterogeneous across eccentric locations. Specifically, the attentional modulations of these neural signals peaked at the parafoveal locations but were reduced as visual stimuli got closer to the fovea or appeared further away from the parafoveal locations towards the peripheral direction. That said, in trials with a relatively higher level of eye movements, the heterogeneous patterns of attentional modulations of the SSVEP and SND data were less robust or even became homogeneous across eccentricities. Together, our findings suggest that sustained visuospatial attention operates differently across different eccentric locations and the contradictory findings in past literature could be due to confounds from differences in the level of eye movement artifacts across studies.

## Results

**Figure 1:**
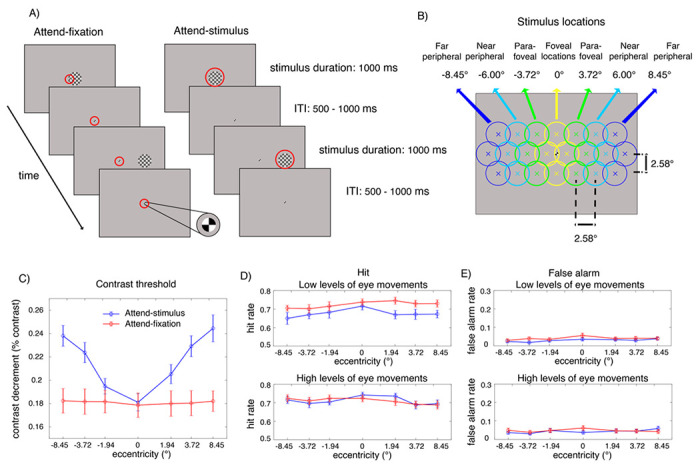
Behavioral tasks and results. (A) The sustained spatial attention tasks. (Left in A) In the attend-fixation task, subjects were instructed to fixate at a small 100%-contrast checkerboard stimulus located at the center of the screen to detect a rare contrast decrement (20% target trials), while ignoring a large 50%-contrast checkerboard stimulus presented at 1 of 22 possible locations: 4 foveal, 6 parafoveal, 6 near peripheral, and 6 far peripheral locations, marked by yellow, green, cyan, and blue circles in (B), respectively. (Right in A) In the attend-stimulus task, subjects covertly attended to the large 50%-contrast checkerboard stimulus that could appear at any of the 22 locations to detect a rare contrast dimming at the stimulus location (20% target trials). Red circles (not physically presented) indicate areas on the screen where subjects were instructed to pay attention to (C) Contrast thresholds plotted as a function of eccentricity in the attend-stimulus and attend-fixation conditions. (D-E) hit and false alarm rates plotted as a function of eccentricity in trials with low and high levels of eye movements, respectively. The error bars in (C-E) represent the within-subject standard errors of the mean (SEMs).

In the present study, we recorded behavioral and EEG responses from human subjects performing a covert visuospatial attention task, where they either attended to a 50%-contrast flickering checkerboard stimulus presented at 1 of 22 possible locations along an elongated hexagonal grid spanning ~ 2.58° x 9.03° visual angle across the entire computer screen or attended to a 100%-contrast central fixation (see detail in Figs. 1A–B and the Materials and Methods section). In the attend-stimulus blocks, they were instructed to fixate at the central fixation, covertly shift their attention to the stimulus location without producing eye movements and sustain their attention at the stimulus location in order to detect a contrast dimming that could occur in 20% of the entire trials. To monitor SSVEPs, the 50%-contrast checkerboard stimulus was flickered at 18.75 Hz for 1,000ms. In order to ensure that subjects sustained their attention throughout the entire stimulus duration, the contrast dimming event could appear any time from 150–650 ms after the stimulus onset for 350ms. Intertrial intervals (ITI) were also jittered from 500-1,000 ms so that subjects could not predict stimulus onset. The stimulus presentation in the attend-fixation blocks was identical to that in the attend-stimulus blocks except that the contrast dimming appeared at the central fixation and subjects were instructed to attend to the fixation throughout the entire block and ignore the presence of the 50%-contrast checkerboard stimulus that could appeared at 1 of the 22 possible locations. Here, the subjects’ task was to detect contrast dimming at the central fixation that could in 20% of the entire trials.

In order to control the level of task difficulty across attention conditions and eccentric locations, we adjusted the degrees of contrast decrement (i.e., contrast thresholds) on a block-by-block basis to maintain hit rate at around 0.7. To do this, we grouped the 22 stimulus locations into 7 sets of eccentricities and adjusted the contrast thresholds separately for the visual stimuli that fell into these individual groups: +8.45°, −3.72°, −1.94°, 0°, + 1.94°, + 3.72°, and + 8.45° visual angle, where 0° is the fovea and − and + values indicate the averaged eccentricity relative to the left and right of the fovea (Fig. 1B). Here we termed stimuli that fell into 0°, ± 1.94°, ± 3.72°, and ± 8.45° eccentricities foveal, parafoveal, near peripheral, and far peripheral stimulations, respectively.

### Behavioral results

As illustrated in Fig. 1C, in the attend-fixation condition, we found that the level of contrast thresholds measured at the fixation did not change significantly as the eccentricity of the competing visual stimulus increased (F(6, 162) = 0.13, p = 0.992). However, in the attend-stimulus condition, the contrast threshold significantly increased as a function of eccentricity (F(6,162) = 26.11, p < 0.001). This result is consistent with the increased receptive field (RF) sizes and decreased cortical magnification factors of neurons in the early visual areas as a function of eccentricity (Cowey and Rolls, 1974; Gattass et al., 1981, 1988; Sereno et al., 1995; Engel et al., 1997; Duncan and Boynton, 2003; Wandell et al., 2007; Dumoulin and Wandell, 2008; Freeman and Simoncelli, 2011; Harvey and Dumoulin, 2011).

The difficulty levels of contrast detection were well maintained at ~ 0.7 hit rate across the different attention conditions and eccentricities (mean hit rate = ± SD = 0.70 ± 0.016) (Fig. 1D). There was a slight but significant main effect of eccentricity on hit rate (range = 0.69–0.73 across all locations; F(6, 162) = 2.80, p = 0.013). Post-hoc paired t-tests show that hit rate in the foveal locations were significantly higher than all parafoveal and peripheral locations (t(27)’s = 2.19–3.22, p’s = 0.003–0.037, two-tailed due to the known direction of the attention effect, Holm-Bonferroni corrected with the threshold of 0.05). However, there was neither a significant main effect of attention F(1, 27) = 3.21, p = 0.085) nor a significant interaction between attention and eccentricity on hit rate F(6, 162) = 0.35, p = 0.912).

**Figure 2:**
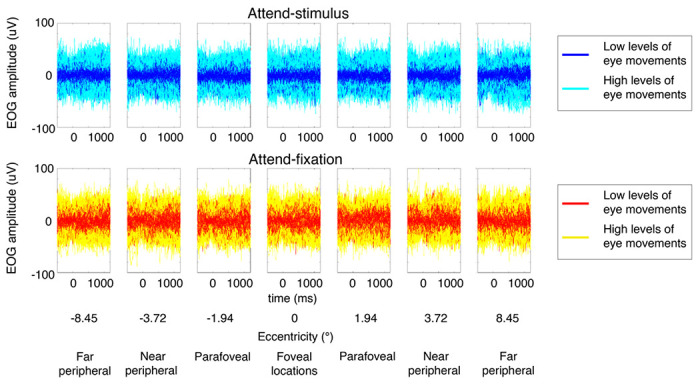
The trial-by-trial EOG signals plotted across all subjects. The EOG data were divided into trials with low vs. high levels of eye movements using the median split method.

To examine the influence of eye movement, we sorted trials based on the level of EOG signals (see Fig. 2 and Materials and Methods). We found that there was a significant interaction between the level of eye movements and attention F(1, 27) = 4.32, p = 0.047). This is driven by a significant main effect of attention in trials with low levels of eye movements (F(1, 27) = 4.77, p = 0.038) but no significant main effect of attention in trials with high levels of eye movements F(1, 27) = 0.02, p = 0.899). Post-hoc t-tests revealed that in trials with lower levels of eye movements, the attention effects on hit rate were significant at the parafoveal locations (t(27) = 2.74, p = 0.005, one-tailed due to the known direction of the attention effect, Holm-Bonferroni corrected with the threshold of 0.0125) and nearly significant at the peripheral locations (t(27)’s = 1.67–1.71, p’s = 0.049–0.053, one-tailed) but not significant at the foveal locations (t(27) = 0.79, p = 0.218, one-tailed). On the other hand, there was no significant attention effect on hit rate at any location for trials with high levels of eye movements (t(27)’s = 0.38–0.87, p’s = 0.197–0.354, one-tailed), suggesting that small saccadic responses were necessary for subjects to maintain similar levels of hit rate across eccentricities in the attended-stimulus condition due reduced contrast sensitivity to visual stimuli in the parafoveal and peripheral locations (see the contrast thresholds results where higher degrees of contrast changes were required for subjects to perform the contrast detection task in Fig. 1C).

As illustrated in Fig. 1D, the false alarm rate in general was relatively low (mean false alarm rate± SD = 0.036 ± 0.008). There was a slight but significant eccentricity effect of false alarm rate (range = 0.024–0.048 across all locations; F(6, 162) = 5.37, p < 0.001). That said, there was no significant main effect of attention F(1, 27) = 0.51, p = 0.481) and no significant interactions between attention, eccentricity, and the level of eye movements (F’s = 1.78–1.97, p’s = 0.073–0.194).

### Steady-state visually evoked potential (SSVEP) results

To monitor the effect of sustained visual attention on the stimulus-evoked neural activity in the early visual cortex, we measure SSVEPs, which were the phase-locked visually evoked EEG responses that oscillated at the same frequency as of the frequency of the flickering visual stimulus (i.e., 18.75 Hz). We used the SSVEP technique here because it is thought to capture the population-level visually evoked responses generated from the early visual areas (reviewed in Refs [58–59]; also see Refs (Regan, 1989; Muller et al., 1997; Di Russo et al., 2007; Andersen et al., 2008, 2012; Fuchs et al., 2008; Andersen and Muller, 2010; Störmer et al., 2013, 2014; Itthipuripat et al., 2013, 2014b, 2018, 2019)). Moreover, many past studies have consistently shown that SSVEP is a sensitive measure for neural gain in many variants of sustained visual attention tasks (Müller et al., 1998, 1997; Di Russo et al., 2007; Andersen et al., 2008, 2012; Fuchs et al., 2008; Andersen and Müller, 2010; Störmer et al., 2013, 2014; Itthipuripat et al., 2013, 2014a, 2018, 2019).

**Figure 3:**
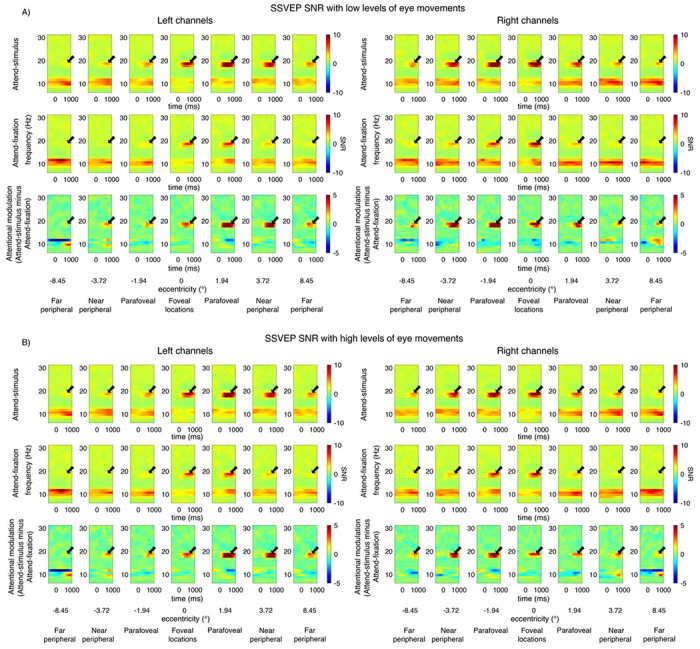
The event-related time frequency plots of the steady-state visually evoked potential (SSVEP) data across different attention conditions and eccentric locations from the left and right posterior occipital electrodes in trials with low vs. high levels of eye movements based on the EOG data in Figure 2. The SSVEP signals peaked at the stimulus flicker frequency (i.e., 18.75 Hz) as indicated by the black arrows.

**Figure 4:**
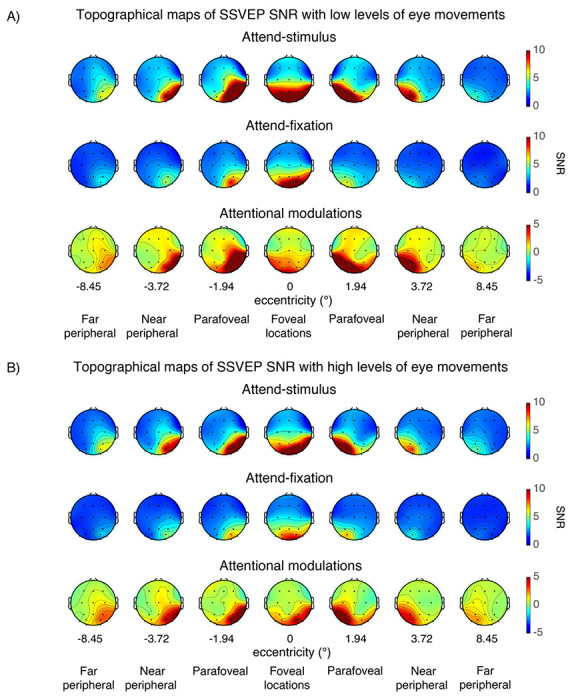
The topographical maps of the steady-state visually evoked potential (SSVEP) data (averaged from 300-1,000 ms post-stimulus onset) across different attention conditions and eccentricities in trials with low vs. high levels of eye movements based on the EOG data in Figure 2.

**Figure 5:**
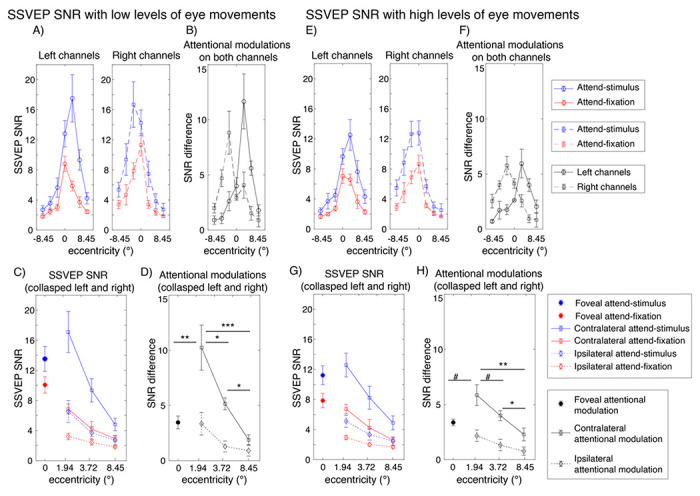
Attentional modulations of the SSVEP signals across eccentricity in trials with low and high levels of eye movements (A-D and E-H, respectively). (A) SSVEP SNR in the attend-stimulus and attend-fixation conditions measured from the left and right posterior occipital electrodes plotted as a function of eccentricity in trials with low levels of eye movements (B) The difference plot showing the degrees of attention modulations (attend-stimulus minus attend-fixation) from both sets of electrodes plotted in (A). (C) Same as (A) but the data were collapsed across the left and right electrodes. (D) Same as (B) but the data were collapsed across the left and right electrodes. (E-H) Similar to (A-D) but the data were obtained from trials with high levels of eye movements. The error bars in all sub-figures represent the within-subject standard errors of the mean (SEMs). #, *, **, and *** show marginal and significant differences in attentional modulations across eccentric locations with p’s < 0.1, 0.05, 0.01 and <0.001, respectively (two-tailed).

Consistent with previous reports (Möller et al., 1998, 1997; Di Russo et al., 2007; Andersen et al., 2008, 2012; Fuchs et al., 2008; Andersen and Müller, 2010; Störmer et al., 2013, 2014; Itthipuripat et al., 2013, 2014a, 2018, 2019), we observed robust SSVEP signals peaking at the flicker frequency of 18.75Hz and the spectral power at the SSVEP frequency peaked at the posterior occipital electrodes (Figs. 3–4). For the visual stimuli appearing within the fovea, the SSVEP signals were distributed bilaterally at the posterior occipital sites. However, as the stimuli were presented at the parafoveal and peripheral locations, the SSVEP signals shifted towards the contralateral compared to the ipsilateral electrodes, resulting in a significant interaction between eccentricity and channel location (i.e., left and right posterior occipital electrodes) (F(6,162) = 23.97, p < 0.001) (see Fig. 5). Overall, the mean SNR of the bilateral SSVEP signals elicited by the foveal stimuli were comparable to those of the contralateral SSVEP signals elicited by the parafoveal stimuli (t(27) = −0.39, p = 0.698, two-tailed, not passing the Holm-Bonferroni-corrected threshold of 0.05). However, the SNR of the contralateral SSVEP signals reduced significantly as the visual stimuli appeared in the eccentric locations further in the periphery, resulting in a significant main effect of eccentricity on the SSVEP SNR (F(6,162) = 34.49, p < 0.001). Post-hoc paired t-tests showed that the SSVEP SNR for the foveal and parafoveal locations were significantly higher than those for the near and far peripheral locations (t(27)’s = 4.28–7.06, p’s < 0.001, two-tailed). In addition, the SSVEP SNR was significantly higher for the near compared to the far peripheral locations (t(27) = 4.30, p < 0.001, two-tailed, all tests passed the Holm-Bonferroni-corrected threshold of 0.025). Taken together, these results were consistent with the fact that foveal vision has higher cortical magnification than peripheral vision (Cowey and Rolls, 1974; Sereno et al., 1995; Engel et al., 1997; Duncan and Boynton, 2003; Wandell et al., 2007; Harvey and Dumoulin, 2011).

As expected, attention significantly increased the SNR of the SSVEP signals (F(1,27) = 18.17, p < 0.001). Importantly, there were significant interactions between attention and eccentricity (F(6,162) = 7.23, p < 0.001) as well as between these factors and channel locations (F(6,162) = 8.62, p < 0.001), showing that the pattern of attentional modulations of SSVEP data was heterogeneous across eccentricities (Figs. 5A–D). As illustrated in Figs. 5D and 5H, these interactions could be described by two characteristics of attentional gain patterns of the SSVEP results across eccentricities: (i) the reduction of attentional modulations of the SSVEP signals at the foveal compared to the parafoveal locations (t(27) = −2.85, p = 0.008, two-tailed) and (ii) the reduction of the attentional modulations at the more peripheral direction compared to the parafoveal locations that occurred in a graded fashion (t(27) = 3.59, p = 0.001 for far peripheral vs. parafoveal location; t(27) = 2.90, p = 0.007 for far vs. near peripheral locations, all tests were two-tailed and passed the Holm-Bonferroni-corrected threshold of 0.025).

The SNR of the SSVEP signals also depends on the level of eye movements as the SSVEP SNR was significantly higher in trials with low compared to high levels of eye movements (F(1,27) = 21.6, p < 0.001) (Compare Figs. 5A and 5C to 5E and 5G). Importantly, the heterogeneous pattern of attentional modulations across eccentricities reported above was relatively more robust in trials with low levels of eye movements compared to those with high levels of eye movements, resulting in a significant three-way interaction between attention, eccentricity, and the level of eye movements (F(6,162) = 2.44, p = 0.028) (Compare Figs. 5B and 5D to 5F and 5H). As illustrated in Fig. 5D, in trials with low levels of eye movements, there was a significant reduction in the SSVEP SNR at the foveal compared to the parafoveal locations (t(27) = −2.80, p = 0.009, two-tailed). Also, the SSVEP SNR significantly reduced as visual stimuli appeared in the locations peripheral to the parafoveal locations (near peripheral vs. parafoveal location: t(27) = 2.60, p = 0.015; far peripheral vs. parafoveal location: t(27) = 3.97, p < 0.001; far vs. near peripheral locations: t(27) = 3.47, p = 0.002, all tests were two-tailed and passed the Holm-Bonferroni-corrected threshold of 0.05). For trials with high levels of eye movements, there was only a marginal difference between the SNR level of the SSVEP signals across the foveal and parafoveal locations (t(27)’s = −2.04, p = 0.052, two-tailed) (Fig. 5H). The reduction of the SSVEP SNR towards the periphery was significant but relatively less robust as there were significant SNR differences between parafoveal and far peripheral locations (t(27) = 3.55, p = 0.001, two-tailed) and between the near and far peripheral locations (t(27) = 2.38, p = 0.025, two-tailed) but no difference between the parafoveal and near peripheral locations (t(27) = 1.77, p = 0.088, all tests were two-tailed with the Holm-Bonferroni-corrected threshold of 0.025).

Overall, the SSVEP results suggested that the attention effects of early sensory responses as measured by SSVEPs were heterogeneous across eccentricities. Moreover, eye movements affected the SNR level of the SSVEP signals and the robustness of the heterogeneous pattern of attentional modulations of the SSVEP data measured as a function eccentricity.

### Sustained negative deflection (SND) results

Next, we examined the slow negative-going wave in the ERP data, termed here as the sustained negative deflection (SND). The negative deflection in the ERP like the SND component has been found to track the locus of spatial attention in behavioral tasks where the stimulus appeared at the peripheral locations (Grent-’t-Jong and Woldorff, 2007; Kelly et al., 2009; Itthipuripat et al., 2018, 2019; Hakim et al., 2019). Specifically, these studies found that attending to the peripheral locations increased the amplitude of the negative deflection (i.e., it becomes more negative) in the contralateral posterior occipital electrodes (Itthipuripat et al., 2018, 2019; Hakim et al., 2019). Importantly, a seminal study combining EEG and fMRI demonstrated that the SND component reflects the top-down attentional biasing signals from the frontoparietal cortex onto the upstream visual areas (Grent-’t-Jong and Woldorff, 2007).

**Figure 6:**
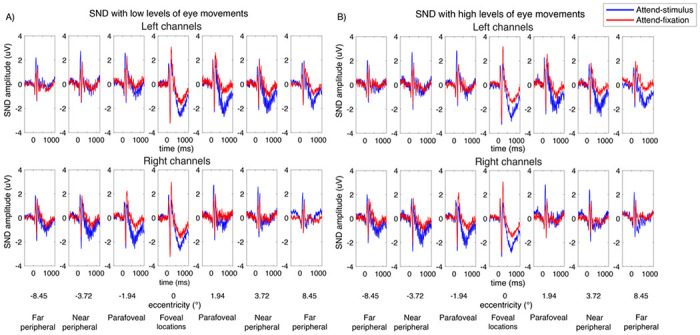
SND traces across different attention conditions and eccentricities from the left and right posterior occipital electrodes in trials with low vs. high levels of eye movements based on the EOG data in Figure 2. The shading areas represent the within-subject standard errors of the mean (SEMs).

**Figure 7:**
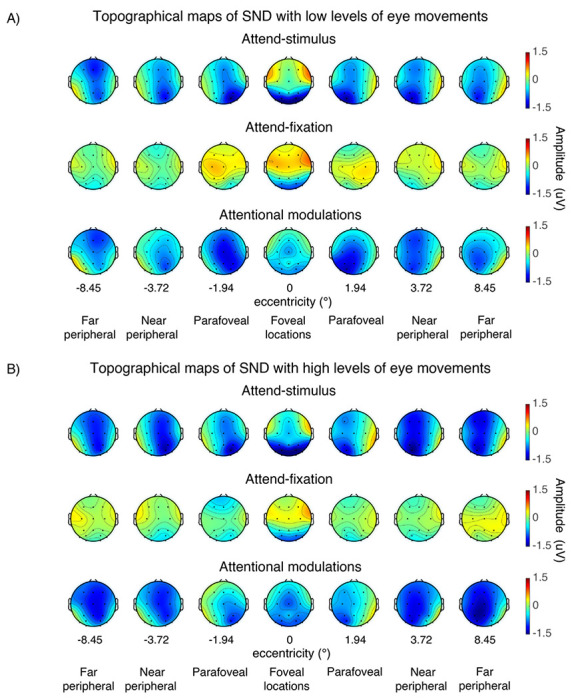
The topographical maps of the SND amplitudes (averaged from 300-1,000 ms post-stimulus onset) of the data shown in Figure 6.

**Figure 8:**
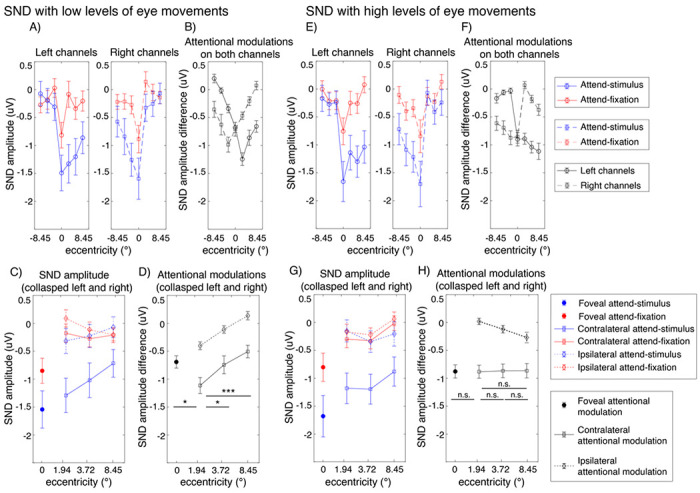
Attentional modulations of the SND amplitudes across eccentricity in trials with low and high levels of eye movements (A-D and E-H, respectively). (A) The SND amplitudes in the attend-stimulus and attend-fixation conditions measured from the left and right posterior occipital electrodes plotted as a function of eccentricity in trials with low levels of eye movements (B) The difference plot showing the degrees of attention modulations (attend-stimulus minus attend-fixation) from both sets of electrodes plotted in (A). (C) Same as (A) but the data were collapsed across the left and right electrodes. (D) Same as (B) but the data were collapsed across the left and right electrodes. (E-H) Similar to (A-D) but the data were obtained from trials with high levels of eye movements. The error bars in all sub-figures represent the within-subject standard errors of the mean (SEMs). * and *** show significant differences in attentional modulations across eccentric locations with p’s < 0.05 and <0.001, respectively (two-tailed). n.s. = nonsignificant.

In the present study, we found the SND component arising from ~ 300ms to 1000ms after the stimulus onset (Figs. 6–7). For the parafoveal stimuli, the SND were distributed centrally at the posterior occipital electrodes and moved more contralaterally as the locations of the stimuli were further away from the fovea. As illustrated in Fig. 8, we observed the higher bilateral SND amplitude (i.e., more negative) elicited by the parafoveal stimulation compared to the contralateral SND amplitude elicited by the more peripheral locations, resulting in a significant interaction between eccentricity and channel location (left and right posterior occipital electrodes) (F(6,162) = 18.72, p < 0.001). Post-hoc paired t-tests showed that the SND amplitudes for the foveal locations were significantly more negative than those in the more peripheral locations (t(27)’s = 2.24–3.36, p’s = 0.002–0.034, two-tailed, passing the Holm-Bonferroni-corrected threshold of 0.05). Additionally, the SND amplitudes in the far peripheral locations were significantly less negative than those in the near peripheral location (t(27) = −2.81, p = 0.009, two-tailed, passing the Holm-Bonferroni-corrected threshold of 0.0167) but the SND amplitudes in the parafoveal location did not differ from those in the far peripheral locations (t(27)’s = 0.49–1.61, p’s = 0.120–0.631, two-tailed). Together, these data suggested that the amplitude reduction of the SND component towards the peripheral direction occurred in a graded fashion.

Similar to the SSVEP results, we observed significant interactions between attention and eccentricity (F(6,162) = 2.94, p = 0.009) as well as between these two factors and channel location on the SND amplitudes (F(6,162) = 27.88, p < 0.001). Importantly, we found a significant three-way interaction between the level of eye movement, attention, and eccentricity, suggesting that the patterns of attentional modulations of the SND amplitudes across eccentricity were different between trials with the low and high levels of eye movements. Specifically, in trials with low levels of eye movements, we found the heterogeneous pattern of attentional modulations of the SND amplitudes similar to the SSVEP results (Figs. 8B and 8D). That is there was a significant reduction of attentional modulations of the SND amplitudes at the foveal compared to the parafoveal locations (t(27) = 2.45, p = 0.021, two-tailed) and the degree of attentional modulations of the SND amplitudes also decreased significantly in the peripheral compared to the parafoveal locations (near peripheral vs. parafoveal location: t(27) = −2.25, p = 0.033 ; far peripheral vs. parafoveal location: t(27) = −3.76, p < 0.001, two-tailed). In contrast, we found that the attentional modulations of the SND amplitudes were comparable across all eccentric locations in trials with high levels of eye movements (t(27)’s = 0.01–0.11, p’s = 0.911–0.990, two-tailed) (Figs. 8F and 8H).

Taken together, the SDN data suggested that the degrees of attentional modulations of the top-down attention signals from the frontoparietal regions also differed across eccentricities when eye movements were carefully controlled and the observed heterogeneity of the pattern of attentional modulations of the SDN signals depend on the level of eye movement.

## Discussions

Despite the fact that foveal and peripheral visions have distinct neural architectures and functions, it has been debated if attention operates similarly or differently across eccentricities. We found that the patterns of attention modulations of both early sensory responses as indexed by the SSVEPs and the frontoparietal neural activity as indexed by the SND component varied depending on eccentric locations. In trials with a negligible level of eye movement, we found that the degrees of attentional modulations of SSVEPs and SND were maximal at the parafoveal locations. Interestingly, the degrees of attentional modulations of these neural signals reduced at the locations more foveal or more peripheral to the parafoveal locations, resulting in the heterogeneous pattern of the attention effects across different eccentricities. Importantly, the heterogeneity of attentional modulations of the SSVEP and SND data were relatively less robust in trials with higher levels of eye movements. Together, these results suggest that sustained visuospatial attention operates differently across different eccentricities and the effects of covert visuospatial attention on early sensory responses and frontoparietal signals depend on the level of eye movements.

The reduction of the attentional modulations of the SSVEP at the locations in our present study were consistent with a previous single-unit recording study, which found the reduced spatial summation and attentional gain of neurons in areas near the fovea of the primary visual cortex in macaque monkeys (Roberts et al., 2007). The similarity between our SSVEP results in humans and the previous single-unit results in monkeys suggests that the way that attention affects early sensory processing near the fovea was highly preserved across the two primate species. In contrast to the results reported by the previous monkey study (Roberts et al., 2007), a recent EEG study has argued that the same gain mechanism underlies the effects of attention on the foveal and peripheral vision in humans (Frey et al., 2010). They found the robust attentional modulations of the early visually evoked potential (VEP) were observed when visual stimuli were presented near the fovea (Frey et al., 2010). Note that even though they found significant effects of attention on the VEP evoked by the stimuli near the fovea, they did not systematically compare the results across different eccentric locations. Thus, this single demonstration is insufficient for concluding that attention operates via similar gain mechanisms across eccentricities.

The reduction in the attentional modulations of the SSVEP (in the present study) and single-unit data at the fovea could be due to the possibility that the ignored stimuli at the fovea can compete for more attentional resources than the ignored stimuli at more peripheral locations. This may be because of the inevitable overlap between the ignored stimulus and the attentional field at the central fixation. Consistent with this idea, we found a similar reduction in the attentional modulations at the fovea for the SND component, which has been used to index the influence of top-down control signals from the frontoparietal cortex onto the upstream visual areas in the occipital cortex (Grent-’t-Jong and Woldorff, 2007). Note that the reduced attentional modulations at the fovea observed in our study should not be influenced by the saturation of the SSVEP and SND signals because the stimuli were presented at 50% contrast.

As the eccentric locations of attention and visual stimuli were further away from the parafoveal locations in the peripheral direction, the attentional modulations on the SSVEP and the SND signals also decreased. This is consistent with the behavioral results where contrast sensitivity and behavioral accuracy decreased as a function of eccentricities in the attend-stimulus compared to the attend-fixation conditions in trials with low levels of eye movements. Based on past studies, differences in task difficulty could lead to varying degrees of attentional gain modulations in the early visual cortex (Spitzer et al., 1988; Spitzer & Richmond, 1991; Chen et al., 2008; Motter, 1993; Boudreau et al., 2006; Handy and Mangun, 2000; 2001; Prinzmetal et al., 2009; Sawetsuttipan et al., 2023). Driven from this idea, the increase in perceptual difficulty at processing visual stimuli in the periphery could contribute to this reduction in the attentional modulations of the SSVEP and SND signals observed in the present study.

The similar decreases in the attentional modulations in the periphery were observed in the fMRI data measured in several regions within the early visual, ventral, and lateral occipital areas (Bressler et al., 2013).The drop-off of the attention-related neural signal in the periphery has also been reported by a recent EEG study that measured the N2pc, the contralateral-vs-ipsilateral ERP difference, commonly known to index target selection processes (Papaioannou and Luck, 2020). The differences between our SND and the recent N2pc results were that the SND modulations sustained over a much longer period of time and that the SND data were obtained from the non-target presented trials while the N2pc data were directly related to the targets. Therefore, the observed modulations in our SND data reflected the effects that sustained attention had purely on sensory processing and were not influenced by any target- or response-related processes.

In trials with a relatively higher level of eye movements, the overall SNR of the SSVEP signals decreased and the heterogeneous pattern of attentional modulations of the SSVEP signals became less robust. We believe this was due to the possibility that eye movements reduced spatial overlaps between stimulus presentations across different trials resulting in the decrease in synchronous activity indexed by the SSVEP signals. While the significant heterogeneous pattern of attentional modulations was still observed in the SSVEP data, the degrees of attentional modulations of the SND data were comparable across eccentricities in trials with the higher level of eye movements. These findings suggest that the effects of covert visuospatial attention on SSVEPs are less susceptible to eye movement artifacts than those of ERP measurements like the SND component. That said, distinct patterns of attentional modulations of the SSVEP and SND signals in trials with varying degrees of eye movements suggest that these neural signals and EOG could be integrated to develop a hybrid BCI system that can track covert visuospatial attention with a precise eye movement control (e.g., Xu et al., 2013; Duszyk et al., 2014; Loughnane et al., 2014; Ordikhani-Seyedlar et al. 2014; Egan et al., 2017; Peng et al., 2020).

In conclusion, our study provided strong evidence suggesting that attention results in the different patterns of visuocortical response modulations across eccentricities, standing in contrast to a recent proposal by Frey and others (2010). These results suggest that sustained visuospatial attention comprises multiple biophysical processes operating differently across different eccentricities, providing new insights how attention augments sensory representations across the entire visual field.

## Methods and Materials

### Subjects

We recruited 40 neurologically healthy human adults who had normal or corrected-to-normal vision from the community surrounding Vanderbilt University, Tennessee to participate in the experiment. Their ages ranged from 18–39 years old. Among these subjects, 31 subjects completed the experimental protocol, which included 2 days of EEG sessions. Prior to their participation, they provided written informed consent as required by the local Institutional Review Board at Vanderbilt University and were compensated at a rate of 10 USD per hour of participation. The data from 2 subjects were excluded from the final analysis because we could not equate task difficulty across eccentricities in these individuals. Specifically, one of them produced spurious false alarms while detecting the target at the furthest peripheral locations (p(FA) = 0.30 and 0.28 for left and right) compared to the foveal locations (p(FA) = 0.04), another subject failed to detect the target at the rightmost peripheral locations (p(hit) = 0.51) compared to the foveal locations (p(hit) = 0.74). Moreover, we excluded the data from another subject because their eye scores based on the EOG data were above 16 uV, corresponding to the deviation of eye movement more than 10° visual angel from the central fixation. These exclusion criteria left the data from 28 subjects in the final analysis and results reported here (15 female, 2 left-handed, mean age = 23.36 ± 4.139 years old).

### Consent statement

The research protocol was approved by the ethics committee of the institutional review board (IRB) at Vanderbilt University and conducted in accordance with the Declaration of Helsinki and the Belmont Report.

### Stimuli and Behavioral Tasks

Stimuli were presented on a Macintosh desktop running MATLAB 7.10.0 (R2010A) (Mathworks Inc., Natick, MA) and the Psychophysics Toolbox: version 3.0.8 (Brainard, 1997; Pelli, 1997). The subjects were seated 80 cm from the CRT monitor with a black background of 1.23 cd/m2 (refresh rate = 75 Hz, 800x600 resolution). The experiment was performed in a dark, sound-attenuated, and electromagnetically shielded room (ETS-Lindgren, Cedar Park, TX, USA).

While EEG signals were being recorded, the participants performed two attention tasks, consisting of attend-fixation and attend-stimulus tasks (Fig. 1A). In the attend-fixation task, the subjects fixated at the small 100% contrast checkered circle (diameter = 0.3° visual angle), while ignoring the larger 50%-contrast checkerboard stimulus (spatial frequency = 1.03 cpd; diameter = 3.06° visual angle) that flickered at 18.75 Hz at one of 22 possible spatial positions on each trial along an elongated hexagonal grid. Note here that the fixation circle did not flicker, and it stayed on the screen throughout the entire experiment. The hexagonal grid consisted of three rows (Fig. 1B). The middle row contained 8 equally spaced spatial positions extending to the left and right of the central fixation along the horizontal meridian (1.29°, 3.87°, 6.45°, 9.03° visual angle to the left and the right of the fixation). The upper row contained 7 equally spaced spatial positions aligned in parallel with the middle row 2.58° above the horizontal meridian. This yielded one stimulus that was placed at 2.58° visual angle above the central fixation and the other 6 stimuli placed at −8.16°, −5.77°, −3.65°, 3.65°, 5.77°, 8.16° visual angle from the central fixation in the upper left (negative values) and upper right quadrants (positive values). The bottom row also consisted of 7 equally spaced spatial positions aligned in parallel with the upper row with 2.58° visual angle below the horizontal meridian. This yielded one stimulus that was placed at 2.58° visual angle below the central fixation and the other 6 stimuli placed at −8.16°, −5.77°, −3.65°, 3.65°, 5.77°, 8.16° visual angle from the central fixation in the lower left (negative values) and upper right quadrants (positive values).

Each block contained 220 trials where the large 50%-contrast flickering checkerboard stimuli could appear at one of these 22 locations (10 repeats of each location) for 1,000 ms, followed by the intertrial interval (ITI) pseudo-randomly drawn from the uniform distribution of 500-1,000 ms. We used 50% stimulus contrast here to avoid response saturation, and past studies have shown robust attentional modulations of behavioral responses and neural activity in the early visual areas when the spatial scopes of attention were relatively broader like in the current design (Reynolds et al., 2000; Martínez-Trujillo and Treue, 2002; Lee and Maunsell, 2009; Reynolds and Heeger, 2009; Herrmann et al., 2010; Itthipuripat et al., 2014b; Zhang et al., 2016).

In the attend-fixation task, subjects were instructed to fixate at the small 100%-contrast checkerboard at the central fixation for the whole block to detect a contrast decrement at the fixation that occurred in 20% of the trials (2 trials for each of the 22 locations of the large flickering 50%-contrast checkerboard stimuli). The contrast decrement at the fixation could occur from 150–650 ms after the onset of the large 50%-contrast flickering checkerboard stimulus, and the constant contrast decrement at the fixation stayed on for 300 ms before returning to the baseline level of 100% contrast. The trial sequence was shuffled so that subjects could not predict trial types and the locations of the to-be-ignored stimuli. We adjusted the levels of the contrast decrement at the fixation block-by-block to maintain hit rates of ~ 0.7. We did this separately for when the large checkerboard stimuli appeared at the fovea (collapsed between 1.29° to the left and right of the fixation and 2.58° below and above the fixation, marked by yellow circles in Fig. 1B; mean eccentricity = 0°), the left/right parafoveal locations (mean eccentricity of ± 3.72° marked by the green circles), the left/right near peripheral locations (mean eccentricity of ± 6.00° marked by cyan circles) and the left/right far peripheral locations (mean eccentricity of ± 8.45° marked by the blue circles).

The attend-stimulus task was identical to the attention-fixation task except that the contrast of the small 100%-contrast checkerboard at the central fixation would not change. However, on 20% of the trials (2 trials for each of the 22 locations), the large 50%-contrast flickering checkerboards that appeared at the parafoveal and peripheral locations would dim slightly for 300 ms anytime from 150–650 s after the stimulus onset. The subjects were instructed to maintain fixation while covertly attending to the large flickering 50%-contrast stimuli at the parafoveal and peripheral locations to detect contrast decrements. The levels of contrast decrements were adjusted block-by-block to maintain hit rates at about 0.7 for all eccentricities (collapsed into 7 locations; see Fig. 1C). For both attend-fixation and attend-stimulus tasks, the subjects had to press a button on the keypad as fast and correctly as possible when they saw the contrast increment. Note that in the attend-stimulus task, there were multiple eccentric locations where we measured contrast discrimination thresholds from, thus it was challenging to use attention cues to guide attention on a trial-by-trial basis. Instead, we manipulated attention by giving subjects different instructions on a block-by-block basis. Each subject completed 8–10 blocks of each task across the 2 days of the experiments (1,760-2,200 trials in total for each task for each subject). We switched between the attend-fixation and attend-stimulus tasks every block, and block sequences (e.g., attend-fixation first or attend-stimulus first) were counterbalanced across subjects. Each block lasted about 6.5 min, and the entire experiment lasted about 2-2.5 hours including EEG preparation on each day.

### Behavioral analysis

We first computed the algebraic means of the hit and false alarm rates for all attention conditions and 7 sets of the eccentricity levels: 1 foveal, 2 parafoveal (left/right), 2 near peripheral (left/right) and 2 far peripheral locations (left/right) (see Fig. 1D and 1E).

First, we use one-way repeated-measures ANOVAs with a within-subject factor of stimulus eccentricities (7 levels: +8.45°, −3.72°, −1.94°, 0°, +1.94°, + 3.72°, and + 8.45° visual angle, where 0° is the fovea and − and + values indicate the averaged eccentricity relative to the left and right of the fovea) on the contrast thresholds separately for the attend-fixation and attend-stimulus conditions. Note that we did not examine the main effect of attention or the interaction between attention and eccentricity on the contrast thresholds because the data between the attend-fixation and attend-stimulus tasks were measured from different sets of visual stimuli that had different sizes and base-line contrast levels (i.e., the small fixation of 100% contrast vs. the much larger checkerboard stimulus of 50% contrast). Thus, the data between the two tasks were expected to be different based on the physical properties of the stimuli and the fixation, and the data should not be compared across attention conditions.

To test if the task difficulty was equated across the different attention conditions and eccentricities, we use 3-way repeated-measures ANOVAs with the within-subject factors of attention (2 levels: attend-fixation vs. attend-stimulus), eccentricity (7 levels: +8.45°, −3.72°, −1.94°, 0°, +1.94°, + 3.72°, and + 8.45° visual angle), and the level of eye movements (2 levels: low vs. high) measured via EOG to test the main effects of and the interaction between these two factors on hit and false alarm rates. We then used 2-way repeated-measures ANOVAs to examine the main effects of attention and eccentricity on hit and false alarm rates separately in trials with low and high levels of eye movements.

### EEG recording

We recorded EEG data from the 10–20 sites, including Fz, Cz, Pz, F3, F4, C3, C4, P3, P4, PO3, PO4, O1, O2, T3, T4, T5, and T6, and a pair of custom sites OL and OR, which were halfway between O1 and T5 and halfway between O2 and T6, respectively. These EEG data were referenced online to the right mastoid. We monitored blinks and vertical eye movements using an electrode placed below the right eye and tracked horizontal eye movements via a pair of external electrodes affixed ~ 1 cm lateral to the outer canthi of the left and right eyes. The impedance of each electrode was kept below 3 k-Ohm. The EEG data were amplified with a gain of 20,000 using an SA Instrumentation amplifier with a bandpass filter of 0.01–100 Hz at the sampling rate of 250 Hz.

### EEG preprocessing

We preprocessed the EEG data using custom MATLAB scripts and EEGLab11.0.3.1b (Delorme and Makeig, 2004). First, we re-referenced the EEG data offline to the average of the left and right mastoid electrodes. Next, we filtered the data using 0.1-Hz high-pass and 55-Hz low-pass Butterworth filters (3rd order). Next, we segmented the continuous EEG data into epochs extending from 2 s before to 2 s after stimulus onset, and the baseline activity averaged from 0-0.2 s before stimulus onset was subtracted from the EEG data. We then performed independent component analysis (ICA) to remove prominent eye blinks (Makeig et al., 1996) and used threshold rejection and visual inspection to reject trials containing saccades, muscle activity, drifts, and other artifacts. This artifact rejection protocol resulted in the removal of 12.28% ± 4.89% SD of trials across the 28 subjects. The threshold rejection method was used here to disregard epochs contaminated by prominent saccades that could be observed on a trial-by-trial basis. The EOG rejection thresholds for individual subjects were adjusted so that the averaged eye score for each attention condition and each eccentricity was below 1.6 uV, which was about 1° visual angle (Luck, 2005). As a further step to minimize potential confounds from residue eye movements on the EEG data, we sorted the data into trials with low and high levels of eye movements using the median split method. Specifically, we obtained the eye score from each trial by computing the absolute value of the difference between the maximum and minimum values of EOG activity and then divided the EEG (and behavioral) data in each attention condition and each eccentricity into trials with low and high levels of eye movements.

### EEG analysis

The artifact-corrected EEG epochs were sorted into 28 bins: 2 attention conditions (attend-stimulus and attend-fixation) x 7 eccentricities (+ 8.45°, −3.72°, −1.94°, 0°, +1.94°, + 3.72°, and + 8.45° visual angle, where 0° is the fovea and − and + signs indicate the averaged eccentricity to the left and right of the fovea) x 2 levels of eye movements (low and high). To minimize confounds from target- and response-related brain processes, we included only correctly rejected non-target trials (i.e., excluding all target trials and non-target trials with false alarms). These data were used for examining the effects of attention, eccentricity, and eye movement as well as their interaction on two neural markers of visual information processing, consisting of the steady-state visually evoked potential (SSVEP) and the sustained negative deflection (SND).

First, we examined the attentional modulations of the SSVEP signals, which were the phase-locked visually evoked EEG responses that oscillated at the same frequency as the frequency of the flickering visual stimulus thought to be generated from the early visual areas (Regan, 1989; Andersen et al., 2012; Norcia et al., 2015). Many past studies have implicated that the attention-induced increases in the SSVEP power/amplitude reflect the attentional gain amplification of the population-level early sensory responses (Müller et al., 1997; Di Russo et al., 2007; Andersen et al., 2008, 2012; Fuchs et al., 2008; Andersen and Müller, 2010; Störmer et al., 2013, 2014; Itthipuripat et al., 2013, 2014b, 2018, 2019).

To obtain SSVEPs, we first averaged the EEG data across trials to obtain the event-related potentials (ERPs) for individual bins. Then, we filtered the data with a Gaussian wavelet function with a 0.1 fractional bandwidth to obtain frequency-domain coefficients from 6.75 to 30.75 Hz in 1 Hz steps. SSVEPs evoked by the individual stimulus flicker frequency of 18.75Hz were then obtained by computing the power of the coefficients at the center of flicker frequency. The signal-to-noise ratio (SNR) of the SSVEPs were then calculated by dividing the power at the flicker frequency of 18.75Hz by the averaged power of surrounding frequencies including 14.75, 15.75, 21.75, and 22.75 Hz. We then collapsed the SSVEP data into 7 sets of stimulus locations following the behavioral analysis (+ 8.45°, −3.72°, −1.94°, 0°, +1.94°, + 3.72°, and + 8.45° visual angle). We focused the analyses on the left and right posterior occipital electrodes where the signals peaked (i.e., PO3, O1 and OL for the left channels and PO4, O2, and OR for the right channels). Since past studies have found that the effect of sustained attention of SSVEPs started at ~ 300 post-stimulus, we averaged the data from 300-1000ms (Müller et al., 1997, 1998). We then plotted the SSVEP SNR from the left and right posterior occipital electrodes across 7 sets of eccentric locations separately for individual attention conditions and trials with low and high levels of eye movements (Fig. 5). We then used a 4-way repeated-measures ANOVAs to test the main effects of attention (2 levels: attend-fixation and attend-stimulus), eccentricity (7 levels: +8.45°, −3.72°, −1.94°, 0°, + 1.94°, + 3.72°, and + 8.45° visual angle), channel location (2 levels: left and right posterior occipital electrodes), and eye movement (2 levels: low and high) as well as their interactions on the SNR of the SSVEP signals.

Since the SSVEP signals were evenly distributed across the left and right channels for the foveal stimulation, we collapsed the data across the two hemispheres. In contrast to the foveal stimulation, the parafoveal and peripheral stimulations produced the SSVEP signals that peaked over the contralateral electrodes. Thus, for all parafoveal and peripheral locations, we analyzed the data from the contralateral and ipsilateral electrodes, separately. Since the data from the left and right stimulations were qualitative similar, we collapsed the contralateral and ipsilateral responses related to the peripheral stimulation to the left and the right of the central fixation, resulting in 3 different sets of eccentricities for the parafoveal and peripheral locations (mean eccentricities = 3.72°, 6.00°, and 8.45° visual angle). Due to this data collapsing step, the contralateral and ipsilateral SSVEP signals related to the parafoveal and peripheral stimuli were obtained from both left and right channels, identical to the sets of electrodes where we obtained the foveal responses. This allowed the same-electrode comparisons between the bilateral SSVEP signals elicited by the foveal stimulation and the contralateral SSVEP signals elicited by the parafoveal and peripheral stimulations which we examined statistically using paired t-tests.

Note that we focused our analysis on SSVEPs instead of other early sensory responses like the P1 component, which typically occur ~ 70-100ms after stimulus onset because a previous study has argued that visual stimulation with a sharp onset could automatically capture attention, and this could potentially mitigate the effects of endogenous attention on the P1 component (c.f., Frey et al., 2010). Instead, the SSVEP signals were continuous visually evoked responses recorded over 1,000 ms throughout the entire stimulus duration and should not be of this particular concern.

In addition to SSVEPs, we examined the effects of attention and eccentricity on the sustained negative deflection (SND). The SND component is a negative-going ERP that has been shown to track the top-down biasing signals from the fronto-parietal cortex onto the occipital cortex (Grent-’t-Jong and Woldorff, 2007; Kelly et al., 2009; Itthipuripat et al., 2018, 2019; Hakim et al., 2019). The mean amplitudes of the SND component for individual attention conditions and stimulus locations were computed by averaging the stimulus-locked ERPs from 300-1,000 ms after stimulus onset across the same sets of left and right posterior occipital electrodes. We then used a 4-way repeated-measures ANOVAs to test the main effects of attention (2 levels: attend-fixation and attend-stimulus), eccentricity (7 levels: +8.45°, −3.72°, −1.94°, 0°, + 1.94°, + 3.72°, and + 8.45° visual angle), channel location (2 levels: left and right posterior occipital electrodes), and eye movement (2 levels: low and high) as well as their interactions on the SND amplitudes. Next, these SND amplitudes were then subjected to the same data collapsing steps and statistically analyses as the SSVEP data. Since we did not have attention cues in our design, we did not focus our analysis on other ERP components such as early attention directing negativity (EDAN), anterior attention directing negativity (ADAN), late directing attention positivity (LDAP), which have been associated with cue-related processes and preparatory attention (Harter et al., 1989; Yamaguchi et al., 1994; Nobre et al., 2000; Jongen et al., 2007; Murray et al., 2011; Itthipuripat et al., 2023).

## Data Availability

The dataset generated and analyzed during the current study are available in the “Sustained attention operates via dissociable neural mechanisms across different eccentric locations” repository, https://osf.io/wphnj/.
